# Multi-Domain CoP Feature Analysis of Functional Mobility for Parkinson’s Disease Detection Using Wearable Pressure Insoles

**DOI:** 10.3390/s25185859

**Published:** 2025-09-19

**Authors:** Thathsara Nanayakkara, H. M. K. K. M. B. Herath, Hadi Sedigh Malekroodi, Nuwan Madusanka, Myunggi Yi, Byeong-il Lee

**Affiliations:** 1 Industry 4.0 Convergence Bionics Engineering, Pukyong National University, Busan 48513, Republic of Korea; thathsara@pukyong.ac.kr (T.N.); kasunkh@pukyong.ac.kr (H.M.K.K.M.B.H.); hadi_sedigh@pukyong.ac.kr (H.S.M.); myunggi@pknu.ac.kr (M.Y.); 2 Digital Healthcare Research Center, Institute of Information Technology and Convergence, Pukyong National University, Busan 48513, Republic of Korea; nuwanv@pknu.ac.kr; 3 Major of Biomedical Engineering, Division of Smart Healthcare, Pukyong National University, Busan 48513, Republic of Korea; 4 Major of Human Bioconvergence, Division of Smart Healthcare, Pukyong National University, Busan 48513, Republic of Korea

**Keywords:** Parkinson’s disease (PD), smart insoles, center of pressure (CoP), Timed Up and Go (TUG) test, machine learning

## Abstract

Parkinson’s disease (PD) impairs balance and gait through neuromotor dysfunction, yet conventional assessments often overlook subtle postural deficits during dynamic tasks. This study evaluated the diagnostic utility of center-of-pressure (CoP) features captured by pressure-sensing insoles during the Timed Up and Go (TUG) test. Using 39 PD and 38 control participants from the recently released open-access WearGait-PD dataset, the authors extracted 144 CoP features spanning positional, dynamic, frequency, and stochastic domains, including per-foot averages and asymmetry indices. Two scenarios were analyzed: the complete TUG and its 3 m walking segment. Model development followed a fixed protocol with a single participant-level 80/20 split; sequential forward selection with five-fold cross-validation optimized the number of features within the training set. Five classifiers were evaluated: SVM-RBF, logistic regression (LR), random forest (RF), k-nearest neighbors (k-NN), and Gaussian naïve Bayes (NB). LR performed best on the held-out test set (accuracy = 0.875, precision = 1.000, recall = 0.750, F1 = 0.857, ROC-AUC = 0.921) using a 23-feature subset. RF and SVM-RBF each achieved 0.812 accuracy. In contrast, applying the identical pipeline to the 3 m walking segment yielded lower performance (best model: k-NN, accuracy = 0.688, F1 = 0.615, ROC–AUC = 0.734), indicating that the multi-phase TUG task captures PD-related balance deficits more effectively than straight walking. All four feature families contributed to classification performance. Dynamic and frequency-domain descriptors, often appearing in both average and asymmetry form, were most consistently selected. These features provided robust magnitude indicators and offered complementary insights into reduced control complexity in PD.

## 1. Introduction

PD is a progressive neurodegenerative disorder and is the second most common such illness after Alzheimer’s disease [1]. In addition to its cardinal motor symptoms of bradykinesia, rigidity, and resting tremors, most patients eventually develop postural instability as the disease advances. Postural instability is characterized by an impaired ability to maintain balance and often manifests as excessive body sway in individuals with PD [1].

One adopted approach to quantify balance control objectively is to analyze the body’s CoP trajectory during stance and gait. The CoP is defined as the point location of the vertical ground reaction force vector, representing the average position of pressure exerted by the body onto the supporting surface [2]. It is a key biomechanical parameter in gait and balance studies, offering insight into how individuals maintain postural stability. CoP is typically measured using force platforms, balance plates, or pressure-sensing insoles, which capture dynamic shifts in load distribution during movement or quiet standing.

**Figure 1:**
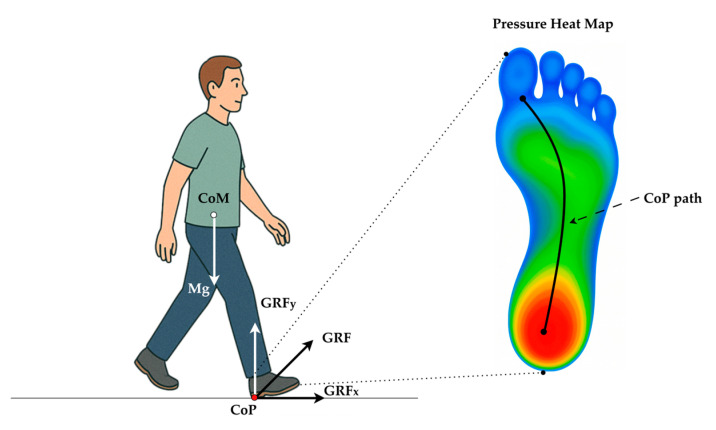
Biomechanical relationship between the body’s CoM and CoP during heel strike (initial contact). Body weight (Mg) acts vertically through the CoM, while the GRF is applied at the CoP under the stance foot and is resolved into horizontal (GRFx) and vertical (GRFy) components. Plantar-pressure heat map (cool → warm colors denote low → high load) with a typical heel-to-toe CoP trajectory (black curve) during normal gait.

Clinically, CoP is a primary mechanism through which individuals actively control their posture by shifting weight anteriorly, posteriorly, or laterally. This is especially important when the Center of Mass (CoM) begins approaching the base of support (BoS) boundaries, such as during perturbed or unstable conditions. While CoM reflects the weighted average position of the body’s mass and is often measured through motion capture systems, CoP is considered the controlling variable that influences the motion of the CoM [3]. This dynamic interaction between CoP and CoM forms the foundation of postural control and balance correction strategies, and is thus critical for understanding motor control mechanisms, especially in populations with neuromuscular impairments such as PD [4]. Figure 1 illustrates the biomechanical interaction between the CoM and the CoP during the stance phase of gait. The body’s gravitational force (Mg) acts vertically downward through the CoM. In contrast, the ground reaction force (GRF) acts upward through the CoP, representing the net point of force application under the foot. Postural adjustments are achieved by shifting the CoP location relative to the CoM, which enables balance correction and stability maintenance during walking or standing. In this context, CoP plays a pivotal role as a biomechanical controller that guides the motion of the CoM to prevent loss of balance.

Abnormal CoP behavior is an early indicator of balance deficits. For example, patients with PD tend to exhibit larger-amplitude sway and higher sway velocities compared to age-matched controls, reflecting the greater effort needed to stabilize their CoM [1]. Consequently, CoP-based metrics (sway path length, velocity, variability, etc.) have been widely used in research to characterize postural instability in PD and other balance-impaired populations [5,6].

**Figure 2:**
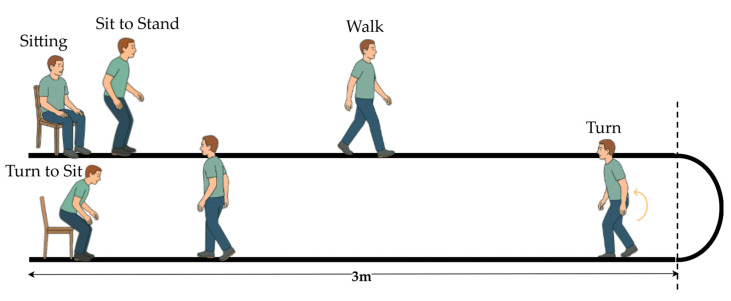
Illustration of the standard TUG test procedure.

The TUG test is a widely used clinical tool for evaluating functional mobility and balance. According to the MDS-UPDRS guidelines, the standard TUG involves a person rising from a chair, walking 3 m, turning around, stepping back, and sitting down [7,8]. Figure 2 illustrates that the standard TUG test comprises multiple functional components reflecting an individual’s dynamic balance and motor coordination.

CoP-based diagnosis, rather than a single sensor type, depends on the setting and purpose. Force platforms during quiet stance in controlled laboratory settings provide high-quality and standardized CoP measurements. For day-to-day clinical use where functional mobility matters, wearable pressure insoles are a strong choice; they directly measure foot-level CoP and asymmetry during different tasks, which are highly informative [9,10]. Smart insoles instrumented with pressure sensors have emerged as a promising technology for gait and balance assessment [11]. These insoles contain arrays of force-sensitive elements distributed across key plantar regions (heel, metatarsals, toes, etc.), allowing continuous measurement of plantar pressure distribution and real-time computation of the CoP under each foot. Wearable pressure insoles enable the capture of gait and postural data outside of specialized laboratories, making monitoring patients in clinical corridors or daily living environments feasible [12].

## 2. Related Works

PD significantly impairs postural control and gait stability, which are essential for safe mobility. The CoP, a biomechanical indicator of plantar pressure distribution, is widely used to quantify these impairments. Studies have consistently shown increased sway magnitude, diminished postural complexity, and altered mediolateral control in PD patients, indicating early motor dysfunction even before clinical symptoms emerge [13,14,15]. With the advent of wearable pressure-sensing insoles, researchers now capture CoP data during both real-world movement and static tasks, enabling the development of machine-learning models for PD-related analyses [16,17].

Extensive research on postural control in PD has highlighted the value of CoP metrics across static and dynamic tasks. For instance, Fernandes et al. [13] reported greater CoP displacement in both anterior–posterior (AP) and medio-lateral (ML) directions in PD patients during quiet standing, particularly under dual-task conditions, even in early-stage PD. Terra et al. [14] demonstrated the high test–retest reliability of CoP features such as the 95% confidence ellipse area and mean sway velocity across seven static balance tasks, especially under eyes-closed Romberg conditions. These findings reinforce the reliability and clinical utility of CoP measures for evaluating and tracking balance impairments in PD [18]. Several studies have aimed to detect subtle postural instabilities in early-stage PD that may not be evident through clinical assessments. Kamieniarz et al. [15] showed that early PD patients exhibit increased low-frequency sway power (0–0.5 Hz) and reduced CoP sample entropy during quiet standing, suggesting more regular and less complex sway patterns. Mid-frequency sway (0.5–1 Hz) in moderate PD also increased. Similarly, Costa et al. [18] reported that PD patients demonstrated greater sway range, speed, and variability than controls, with nonlinear metrics indicating reduced postural complexity. Beyond static tasks, CoP behavior during dynamic activities such as gait initiation has also revealed PD-specific impairments. Bayot et al. [19] found that PD patients, especially those with freezing gait (FOG), adopted a laterally shifted CoP posture before stepping, showed diminished backward CoP shifts during anticipatory adjustments, and experienced prolonged unloading of the swing foot, ultimately resulting in a shorter and slower first step. Engel et al. [6] used convolutional neural networks (CNNs) to analyze frequency-domain CoP and center-of-mass signals from quiet standing, successfully distinguishing PD from controls based on spectral features, despite a small sample size.

Over the past decade, wearable sensor technologies, particularly pressure-sensing insoles, have emerged as valuable tools for monitoring gait and balance in PD beyond traditional lab-based force platform studies [20]. These smart insoles enable real-world assessment of dynamic tasks such as walking and turning, expanding the applicability of CoP and plantar pressure analyses. Mazumder et al. [21] evaluated CoP variation from plantar-pressure data across PD vs. age-matched controls and across mild vs. moderate PD stratified by the Hoehn–Yahr (H&Y) Unified Parkinson’s Disease Rating Scale (UPDRS), and TUG test, highlighting insole-derived CoP metrics as candidate markers of disease progression. Tsakanikas et al. [22] used insoles with 16 pressure sensors and an IMU to compare gait in mild PD vs. healthy older adults, while Ayena and Otis [23] analyzed TUG performance using CoP and gait metrics, revealing slower transitions and increased sway in PD patients. Herbers et al. [16] further evaluated CoP dynamics during static and dynamic tasks in a large cohort of PD patients and controls.

Machine Learning and deep learning methods have also been applied to PD-related analysis using wearable insoles and force platforms. Shalin et al. [17] employed LSTM networks to detect freezing of gait from insole pressure signals, training on time series of CoP coordinates, CoP velocity/acceleration, total GRF, and fraction of GRF from each foot. Recent studies have explored broader diagnostic applications. Jung et al. [24] trained a Transformer-based model on CoP features to classify balance impairment. Fujii et al. [25] applied exploratory factor analysis to 23 quiet-stance CoP variables and then Gaussian mixture model clustering, identifying six subtypes of postural instability in PD that differ in sway magnitude and frequency characteristics. A detailed summary of these studies is presented in Table 1.

Despite advances in measuring gait and balance impairments in PD, many studies still rely on force platform-based assessments during quiet standing or linear walking. Ortega-Bastidas et al. [7] highlighted that 75% of TUG-related studies primarily used inertial sensors to segment the task and extract temporal-positional features such as gait speed and turn duration. However, there is an increasing need to incorporate smart insoles for CoP analysis during complex functions like TUG, which challenge multiple aspects of balance. Wearable smart insoles allow continuous CoP tracking across dynamic, ecologically valid tasks. Most machine-learning studies on PD using insole signals have relied on small, CoP feature sets (often limited to sway magnitude/velocity or a few spectral indices) and have focused on quiet-stance tasks and a narrow dynamic paradigm, typically straight-line walking. According to the literature review performed for the last five years, authors observed that no prior study has evaluated the TUG test in PD using a comprehensive insole-derived CoP feature set spanning all four domains: positional, dynamic, frequency, and stochastic, combined with multi-model machine-learning benchmarking. Addressing this gap, our study utilized a clinically curated, open dataset and an instrumented TUG protocol to extract over a hundred CoP-based features in positional, dynamic, frequency, and stochastic domains for classification, unlike traditional studies focused on only static sway measures. This comprehensive approach enabled us to identify the most robust CoP features and optimal classification models for PD detection. The detailed methodology of the proposed system is presented in the following section.

## 3. Materials and Methods

### 3.1. Datasets and Data Availability

This study utilized data from the WearGait-PD, a recently released open-access dataset [26], a publicly available (dataset last updated on 9 November 2024, accessed for this study on 13 February 2025) and clinically curated repository developed through a collaboration between the U.S. Food and Drug Administration (FDA), the Department of Veterans Affairs (VA), and the Johns Hopkins School of Medicine. For each participant, the dataset includes raw recordings from smart insoles (16 capacitive sensors and a single 6-axis IMU per foot), 11 body-worn 9-axis IMUs, and pressure walkway data.

**Figure 3:**
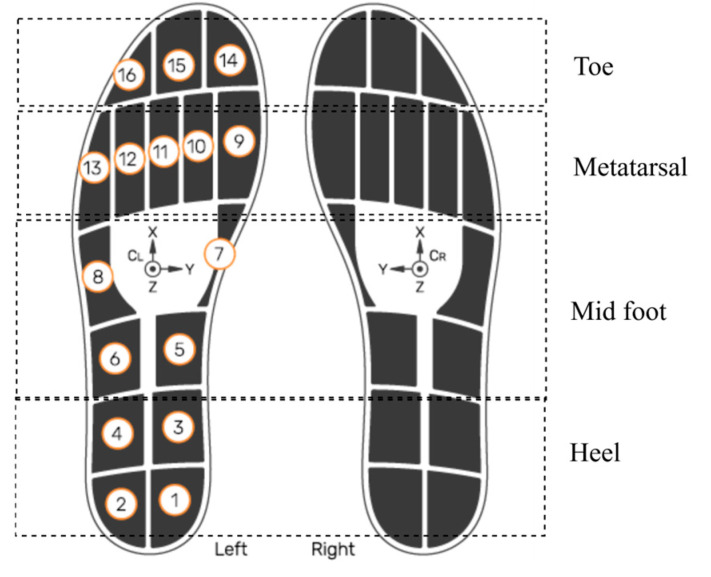
Sensor layout of the smart insole system. Numbered circles (1–16) indicate the manufacturer’s pressure-sensor IDs and their positions. The XYZ axes show the IMU orientation. Smart insole system reproduced with permission from Moticon [27].

This study investigated and utilized smart insole CoP data generated using insole pressure signals. This analysis did not use data from the insole IMU, external IMUs, or pressure walkway. The OpenGo sensor insoles (model—Insole3) (Moticon ReGo AG, Munich, Germany) was used for the data acquisition [27]. As shown in Figure 3, the sensors were strategically embedded across key plantar regions, including the heel (4 sensors), midfoot (4 sensors), metatarsal (5 sensors), and toe region (3 sensors). This dense and anatomically informed distribution ensured high-resolution pressure sampling, which is necessary for accurate CoP estimation. The insoles recorded data continuously at a sampling frequency of 100 Hz, which is adequate to capture dynamic balance adjustments and gait events during the TUG test. According to the manufacturer’s specifications, the pressure sensors provide a measurement range up to 50 N/cm^2^ with a resolution of approximately 0.25 N/cm^2^, allowing precise detection of subtle load shifts. CoP values are internally computed by the Moticon firmware from raw sensor data in the insole’s normalized coordinate system (COS), which has been validated against reference force-platform systems in previous studies [28]. While this precision is generally lower than that of high-end laboratory-grade force platforms, it is sufficient for wearable in-shoe applications. It offers the advantage of portability and real-world usability. Moderate resolution, adequate sampling rate, and anatomically informed sensor distribution enable reliable tracking of load shifts, postural responses, and dynamic stability throughout the TUG task, covering biomechanically critical areas involved in balance and gait transitions [29].

To obtain a consistent and demographically balanced dataset for analysis, we implemented the curation procedure outlined in Algorithm 1. Beginning with the original WearGait-PD dataset ($D_{So}$), participants were screened for completeness of insole recordings during the TUG test. Data streams from non-relevant modalities, including the insole IMU, external IMUs, and the pressure walkway, were systematically excluded to ensure that only plantar pressure signals were retained. This filtering step yielded the intermediate dataset ($D_{TUG}$), which contained valid insole-derived CoP trajectories for all included trials. To mitigate potential group imbalances and ensure fair comparison (PD: 50.64%, Control: 49.35%), we further processed DS_TUG_ to derive a demographically matched subset, $D_{TUG}^{M}$, with approximately equal representation of individuals with PD and healthy elderly controls. The structured procedure formalized in Algorithm 1 provides a reproducible approach to dataset curation, ensuring that subsequent analyses are methodologically rigorous and demographically comparable. Group-wise demographic information is summarized in Table 2.

The procedure for dataset preparation is described in Algorithm 1.
**Algorithm 1:** Dataset Curation for TUG Analysis**Input:** D_so_: WearGait-PD original dataset       M: Demographic metadata (age, group labels)       F: Trial identifiers for TUG tasks **Output:** $D_{TUG}$: Subset with valid insole signals during TUG      $D_{TUG}^{M}$: Demographically balanced subset 1 Initialize $D_{TUG}$ ← ∅; $D_{TUG}^{M}$ ← ∅; 2 **foreach** *participant* p ∈ $D_{So}$ **do** 3   **if** *p contains*
$F_{TUG}$ **AND** *complete insole recordings* **then** 4     Remove modalities {${IMU}_{insole}$*,*
${IMU}_{external}$*,* Walkway}; 5     **if** insole signals are valid ∧ synchronized **then** 6       $D_{TUG}$ ← $D_{TUG}^{M}$ ∪ {p}; 7 **Demographic Balancing**: 8 Compute $n_{PD}$, $n_{HC}$ from $D_{TUG}$; 9 Construct $D_{TUG}^{M}$ such that:   $\frac{n_{PD}}{n_{PD}+n_{HC}}\approx 0.5064$,   $\frac{n_{HC}}{n_{PD}+n_{HC}}\approx 0.4935$   with matched covariates in M; 10 **return** $D_{TUG}$, $D_{TUG}^{M}$;

### 3.2. Insole-Based CoP Measurement and Data Processing

All participants completed the standardized TUG protocol while wearing smart insoles inside their footwear. The TUG test was executed continuously per the MDS-UPDRS guidelines [8]. From a seated position with the back against a chair with armrests, they stood on the “Go” command, walked 3 m at a comfortable self-selected speed, executed a 180° turn at a taped line, walked back to the chair, and sat down. Use of armrests was allowed as needed; three trials were recorded, and throughout the TUG test, the sensors continuously recorded plantar pressure distributions and CoP values from each of the 16 pressure sensors. In biomechanical gait analysis, the CoP represents the weighted average location of all ground reaction forces acting on the plantar surface. Equations (1) and (2) describes the formula used to calculate CoP coordinates in the x and y directions, respectively [30],

(1) $${COP}_{x}=\frac{\sum_{i=1}^{N}d_{xi}F_{i}}{\sum_{i=1}^{N}F_{i}}$$

(2) $${COP}_{y}=\frac{\sum_{i=1}^{N}d_{yi}F_{i}}{\sum_{i=1}^{N}F_{i}}$$

where,

- $d_{xi}\;$ and $d_{yi}\;$ denote the coordinates of the *i*th pressure sensor along the ML and AP axes, respectively.
- $F_{i}\;$ represents the force measured by that sensor.

This formulation ensures that the CoP reflects the net pressure location under the foot at any time. In this study, CoP values were obtained from the Moticon OpenGo insole system, which internally performs this calculation and exports the CoP in a normalized COS. This COS is defined consistently for both left and right feet, with the ML axis ranging from −0.5 to +0.5 and the AP axis from approximately −0.574 to +0.426 [27]. As the Moticon system normalizes the CoP values during data export, no additional transformation or size-specific correction was applied during collection. In this study, all CoP-based analyses were performed directly using these normalized coordinate values, which ensured consistency across participants regardless of insole size. Consequently, all CoP-based features were derived using the normalized CoP values provided by the manufacturer, without conversion to units.

### 3.3. CoP Density Mapping and Trajectory Computation

To comprehensively analyze plantar pressure distributions during the TUG test, we performed CoP density mapping using normalized CoP data from smart insoles. For each cohort (Control, PD) and each foot, we pooled all available TUG CoP samples and visualized them in that normalized coordinate system. We then converted the resulting CoP point clusters into a smooth spatial-occupancy map using a two-dimensional Gaussian Kernel Density Estimate (KDE), yielding a continuous surface where higher intensities indicate locations where the CoP spent more time. We applied identical KDE smoothing, axis limits, and color scaling across all panels to ensure fair visual comparison between groups and feet.

### 3.4. Feature Engineering from CoP Data

Positional features described the dispersion and geometry of the CoP path in both the AP and ML directions. These included metrics such as mean displacement, root-mean-square sway, maximal distance, and confidence ellipse area, which reflect the physical bounds of postural stability (Tables S1 and S2). Dynamic features characterized temporal changes in CoP movement, such as average sway velocity, peak sway velocity, speed variability, and phase plane parameters. These indicators provide insight into the subject’s postural adjustment strategy and neuromotor responsiveness during movement (Tables S3 and S4). Frequency-domain features were calculated from the Power Spectrum Density (PSD) of the CoP signals. Parameters were included, such as mean and centroidal frequency, frequency quotient, and energy content within specific bands (≤0.5 Hz, 0.5–2 Hz, >2 Hz). These capture oscillatory properties of balance regulation and reveal abnormalities in sway dynamics (Table S5). Stochastic features were based on stabilogram diffusion analysis, diffusion scaling coefficient, and critical time. These included critical displacement, critical time, diffusion coefficients, and fractal dimension, providing information about the complexity, predictability, and adaptability of the CoP trajectory under different motor demands (Table S6) [31].

**Figure 4:**
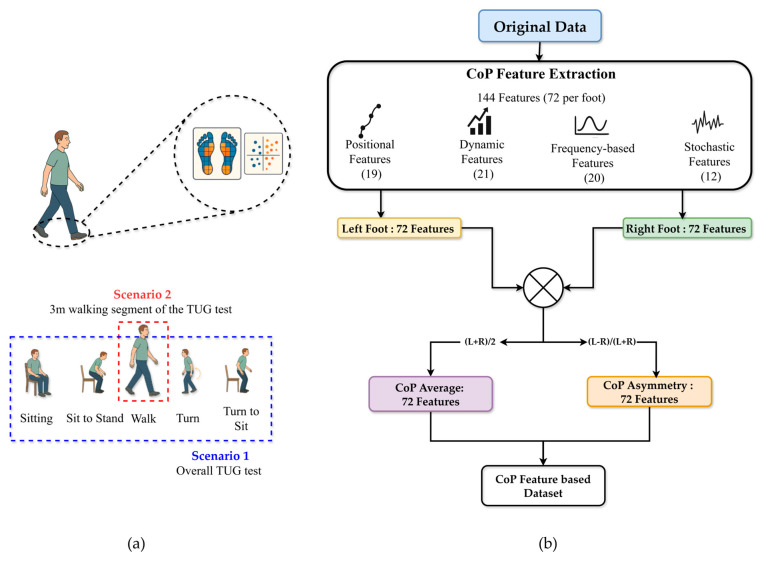
Overview of CoP feature extraction process for classification (**a**) Two scenarios used for evaluation (**b**) CoP feature extraction process.

Two experimental scenarios were analyzed: Scenario 1, which considered the overall TUG test performance, and Scenario 2, which focused specifically on the 3 m walking segment of the TUG test (See Figure 4a). For each participant, CoP features were computed separately for the left and right feet for both scenarios. The feature equations and definitions were adapted from Quijoux et al. [31], with detailed formulations provided in Supplementary Materials. All features were computed per foot using the authors’ open-source implementation. Consequently, 72 features were derived per foot (144 per scenario), encompassing positional, dynamic, frequency-based, and stochastic descriptors. Specifically, two summary measures were calculated for each feature: the average value across both feet and the asymmetric index. As described in Equation (3), the CoP average features (${Avg}_{CoP}$) was calculated as the arithmetic mean of the left and right foot values:

(3) $${Avg}_{CoP}=\frac{{CoP}_{Left}+{CoP}_{Right}}{2}$$

As described in Equation (4), the CoP asymmetry features (${Asy}_{CoP}$) was calculated using the normalized absolute difference between the two sides, capturing gait imbalance:

(4) $${Asy}_{CoP}=\frac{{CoP}_{Left}-{CoP}_{Right}}{{CoP}_{Left}+{CoP}_{Right}}$$

This process yielded 72 average and 72 asymmetrical features, resulting in 144 total features per scenario (see Figure 4b).

### 3.5. Model Development and Evaluation

**Figure 5:**
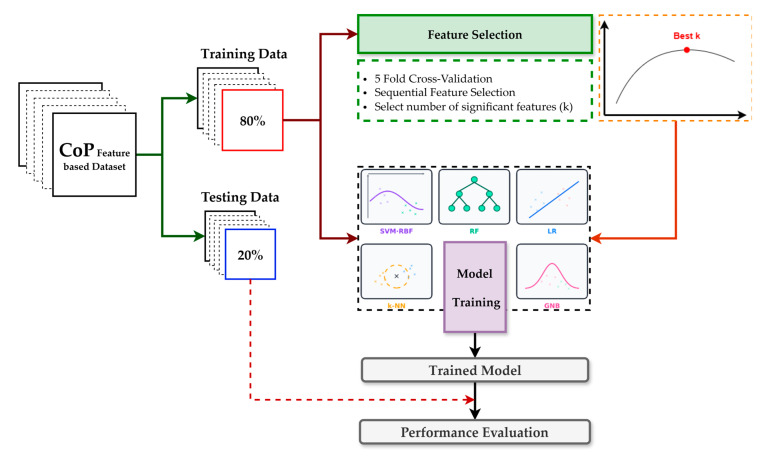
Overview of the model training and performance evaluation pipeline for the classification.

Figure 5 summarizes the protocol. We created a single participant-level 80/20 train–test split and reused this identical partition across all models to ensure fair comparability. Model development was performed exclusively on the training set using a uniform pipeline, sequential forward selection embedded in 5-fold inner cross-validation scored by F1, and then the classifier. The number of selected features was chosen by grid search over *k* ∈ [1, 36] (≤25% of the original 144) to improve stability and interpretability, reduce runtime, and mitigate overfitting. After fixing best *k*, we refit the pipeline on the complete training set and evaluated once on the untouched test set, reporting accuracy, precision, recall, F1, ROC–AUC, and the confusion matrix, alongside ROC and precision–recall. The classifier family comprised SVM-RBF, LR, RF, k-NN, and NB; key hyperparameters are listed in Table 3. These models represent a diverse and widely used set of conventional machine learning approaches for biomechanical classification tasks, offering complementary strengths, and have consistently demonstrated exemplary performance in previous studies [32,33,34].

## 4. Results and Analysis

### 4.1. CoP Density Maps and Trajectory Maps Analysis

**Figure 6:**
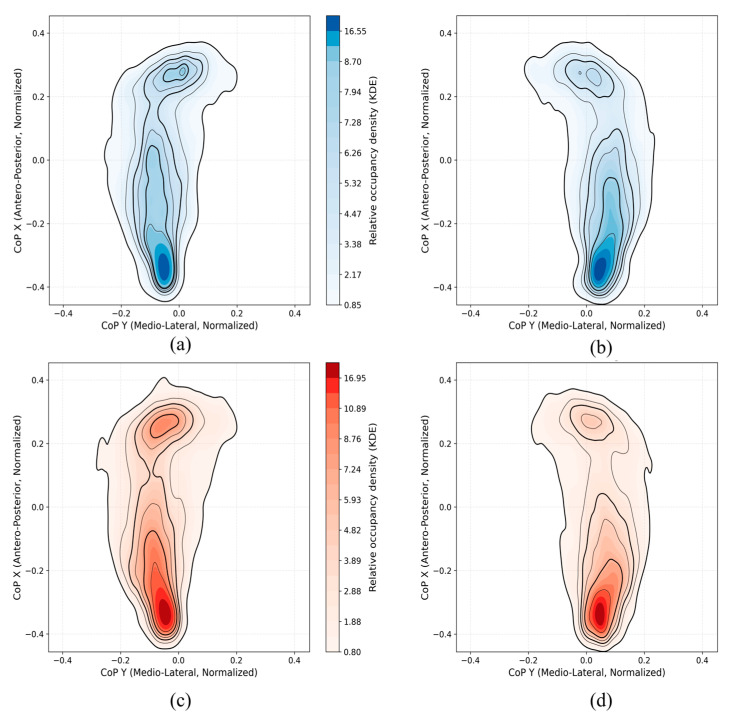
KDE-based CoP density maps illustrating plantar pressure distribution patterns for control participants ((**a**): left foot, (**b**): right foot) and PD participants ((**c**): left foot, (**d**): right foot).

Figure 6 presents the KDE-based CoP density maps for both left and right feet in the control and PD groups. In the control group, the CoP trajectories show concentrated pressure distribution mainly in the heel and metatarsal regions, with relatively balanced density between the two feet. In contrast, the PD group exhibits increased asymmetry between the left and right foot. Specifically, the PD density maps reveal stronger and more localized CoP activity around the heel and forefoot areas, with higher occupancy in one foot. This imbalance is likely due to the typical unilateral motor symptom onset observed in early-stage PD, leading to unequal weight distribution and postural instability. Overall, these visualizations support the idea that PD patients demonstrate distinct and asymmetric plantar pressure patterns compared to controls. The CoP density plots offer a compact visual summary of dynamic balance control and could aid in characterizing subtle gait abnormalities. The control group also exhibited only mild asymmetry in CoP patterns between the dominant and non-dominant legs [35]. This likely reflects normal biomechanical variability.

### 4.2. Machine Learning Classification Performance

**Figure 7:**
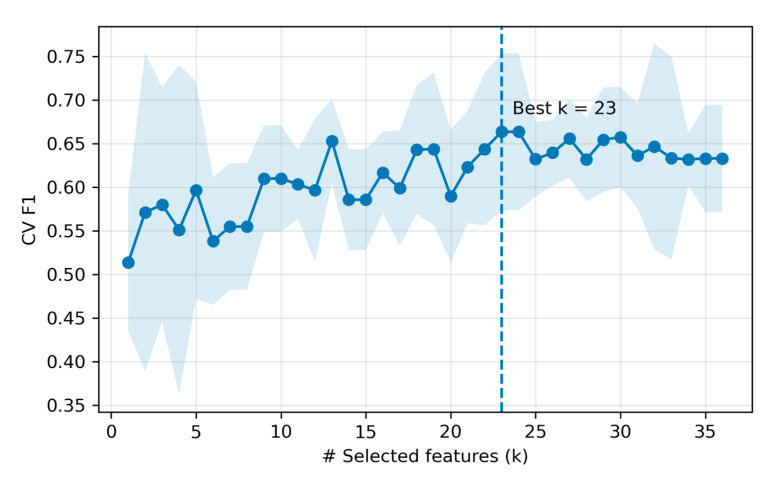
Feature count selection for the top model (LR). The shaded area denotes standard deviation across 5 CV folds at each k.

We compared five classifiers under the fixed protocol on the held-out test set. Overall, LR achieved the highest performance in the test (accuracy = 0.875, precision = 1.000, recall = 0.750, F1 = 0.857; 23 selected features), followed by RF and SVM-RBF of accuracy = 0.812. Per-model test metrics are summarized in Table 4. For the top model, the feature -count selection curve (cross-validation F1 score versus number of features) peaked at k = 23 (inner-CV mean F1 ≈ 0.663), after which performance plateaued (Figure 7). The 23 LR-selected features are listed in Supplementary Materials (Table S7).

**Figure 8:**
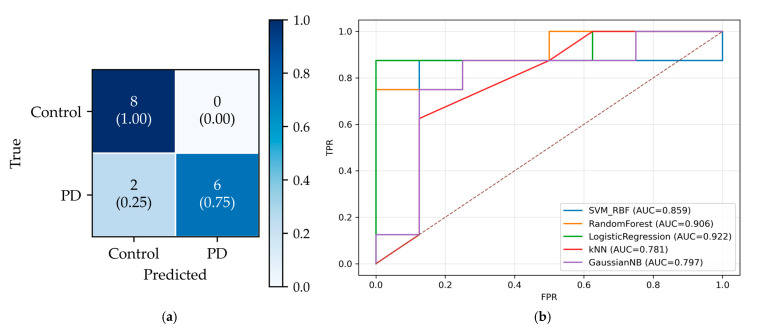
Test performance. (**a**) Confusion matrix for the best model (LR). (**b**) ROC curves for all models on the same test set. The diagonal dashed line shows the baseline, corresponding to AUC = 0.5.

On the held-out total of 16 subjects test dataset (50% PD, 50% controls), the final classifier achieved 14/16 correct predictions. Figure 8a shows the confusion matrix: True Negatives (TN) = 8, True Positives (TP) = 6, False Positives (FP) = 0, False Negatives (FN) = 2, yielding specificity = 1.00 and sensitivity = 0.75. Notably, the chosen operating point produced no false positives (FPR = 0) at the cost of two false negatives (FNR = 0.25). Figure 8b presents the ROC curves, which indicate good class separability for all models (AUC > 0.75). LR achieved the highest AUC (0.922), followed by Random Forest (0.906) and SVM-RBF (0.859). Although the curves have a staircase shape due to the small sample, each lies well above the chance diagonal, confirming meaningful separability.

Table 5 presents the subset of features that sequential forward selection identified in at least three classifiers. The most consistently selected feature was Maximal distance (Radius), retained by all five models, indicating overall sway excursions. A second tier of nine features, each retained by four models, captured complementary aspects of postural control. In the frequency domain, centroidal frequency, Energy content below 0.5 Hz, frequency quotient describes how sway power is distributed across slow versus faster components. Dynamic descriptors mean velocity, mean positive peak velocity, and mean positive peak velocity summarize how CoP moves. Positional features include the principal sway direction and maximal distance. Most consensus features come from the dynamic and frequency families, suggesting the speed and spectral content of CoP motion. Several asymmetry variants were also repeatedly selected, implying that between-limb/lateralized control differences add complementary information, which aligns with the lateralized motor impairment commonly observed in PD. Fewer models retained positive features such as area measures such as 95% confidence ellipse area and range, suggesting that absolute sway magnitude contributes. Notably, one stochastic descriptor the short-term diffusion coefficient, was also repeatedly selected, indicating short latency.

## 5. Discussion

This study investigated the diagnostic potential of CoP-derived features captured from smart pressure insoles during the TUG test to distinguish individuals with PD from age-matched healthy controls. Our machine learning framework revealed that TUG-based CoP features yielded the best classification performance with Sequential Feature Selection. These results highlight the effectiveness of the TUG protocol in revealing PD-specific postural control deficits. To further evaluate the robustness of the proposed approach, we extended the same feature engineering classification pipeline for scenario 2. Interestingly, the classification performance on walking data was consistently lower than for TUG, despite identical preprocessing, feature extraction, and selection methods. As summarized in Table 6, the K-NN classifier on walking data achieved an accuracy of 0.688, F1-score of 0.615, and ROC-AUC of 0.734, substantially below its performance on TUG. These findings indicate that the TUG task’s more dynamic and multi-phase structure provides greater classification capability than straight-line walking when using CoP-derived features alone.

The superior performance of the TUG-based models is physiologically justified. The TUG test integrates multiple functional sub-tasks such as sit-to-stand, walking, turning, and turn-to-sit, placing higher demands on postural control, anticipatory adjustments, and motor coordination. In contrast, straight walking represents a more stable and repetitive task, offering fewer opportunities to capture subtle motor impairments than TUG associated with PD. This is further supported by the analysis of features selected through Sequential Feature selection for the TUG task, which were dominated by average and asymmetry indices of CoP features. These features are closely linked to impaired sway regulation, reduced neuromotor complexity, and lateralized instability in PD.

Under an identical processing pipeline, CoP features derived from the TUG task consistently outperformed those extracted from straight walking in classifying PD versus control participants. Notably, the features repeatedly selected across models were not limited to a single category. Instead, descriptors from positional, dynamic, and frequency domains were frequently retained, indicating that diagnostically relevant information is distributed across both temporal and spectral dimensions of postural sway. By leveraging this multi-domain feature space alongside a rigorous, model-agnostic feature selection strategy and comprehensive evaluation on a dataset, this study moves beyond prior work that focused on limited sway metrics or task-specific sensing modalities. The resulting pipeline offers a reproducible and scalable framework for insole-based CoP analysis during TUG, contributing a valuable tool for early PD assessment and future clinical applications.

Despite promising findings, several limitations should be acknowledged in this study. The classification models utilized were primarily classical machine learning algorithms, and exploration of advanced deep learning approaches might further enhance diagnostic accuracy. Additionally, the absence of explicit segmentation of TUG sub-phases may overlook phase-specific postural instability characteristics, suggesting potential gains from analyzing these segments independently in future research. Future studies may enhance model performance by incorporating explicit TUG phase segmentation, enabling phase-specific feature extraction and interpretation. Moreover, combining CoP features with complementary signals, such as insoles or body-worn inertial measurements, may provide a more comprehensive representation of neuromotor control.

## 6. Conclusions

This study demonstrates that CoP-derived features from wearable smart insoles during the TUG test can distinguish individuals with PD from age-matched controls under a fixed, participant-level protocol. Using sequential forward selection with five-fold cross-validation on the training set, the best test performance was achieved by LR (accuracy = 0.875, F1 = 0.857, ROC-AUC = 0.922) with an optimally selected subset of 23 features. At the same time, RF and SVM-RBF performed slightly lower on the same split. Models trained on the complete TUG task consistently outperformed those trained on the straight 3 m walking segment (k-NN: accuracy = 0.688, F1-score = 0.615, and ROC-AUC = 0.734), underscoring the diagnostic value of functionally rich, multi-phase activities for revealing PD-specific balance deficits. Domain-wise, dynamic, and frequency domain descriptors, often expressed as average and asymmetry indices, were most consistently retained across classifiers, indicating that sway speed and spectral content capture salient PD-related control changes and lateralized instability. Positional measures provided robust magnitude markers of postural excursion; notably, maximal distance (Radius) was selected by all five models, reflecting larger sway envelopes in PD.

Our results support instrumented TUG testing with pressure-sensing insoles as a practical, non-invasive, and scalable approach for screening and monitoring PD motor dysfunction. Future work should validate generalization in larger cohorts, examine phase-specific modeling within TUG (e.g., turns and transitions), and assess portability across devices and clinical settings.

## Figures and Tables

**Table 1:** Summary of prior studies analyzing gait or balance assessment in PD and related disorders.

Study	Year	CoP Features Used	TUG Test Used	Data Collection Method	Machine Learning Used
Fernandes et al. [13]	2015	✔	✘	Pressure platform	✘
Terra et al. [14]	2020	✔	✘	Force platform	✘
Kamieniarz et al. [15]	2021	✔	✔	Force platform	✘
Costa et al. [18]	2024	✔	✘	Force platform	✘
Bayot et al. [19]	2022	✔	✘	Force platform	✘
Engel et al. [6]	2025	✔	✘	Balance board (Force platform)	✔
Jung et al. [24]	2024	✔	✘	Pressure-sensing treadmill	✔
Fujii et al. [25]	2025	✔	✘	Force platform	✔
Herbers et al. [16]	2024	✔	✘	Smart Insole	✔
Ayena & Otis [23]	2022	✔	✔	Smart Insole	✘
Tsakanikas et al. [22]	2021	✔	✔	Smart Insole	✘
Shalin et al. [17]	2021	✔	✘	Smart Insole	✔
Mazumder et al. [21]	2018	✔	✔	Smart Insole	✘

Note: ✔ indicates inclusion of the feature, test, or method, whereas ✘ indicates non-inclusion.

**Table 2:** Group-wise demographic characteristics of participants included in the study.

	PD	Control
Sample size	39	38
Age (Years)	69.03 ± 5.3	73.58 ± 4.6
Gender (Male/Female)	25/14	16/22
Years with PD	7.3 ± 6.0	Not applicable
Weight (kg)	76.83 ± 16.7	80.20 ± 16.9
Height (cm)	173 ± 9.4	168 ± 9.7

**Table 3:** Model-specific hyperparameters were applied during the training process.

Model	Hyperparameters
SVM-RBF	c=1.0, γ =“scale”, class weight = “balanced”, probability = *True*, random state = 42
RF	$n_{estimators}=100$, class weight = “balanced”, random state = 42
LR	solver = “lbfgs”, max_iter = 2000, class weight = “balanced, random state = 42
k-NN	Defaults: n neighbors = 5, weights = ‘uniform’, metric = ‘minkowski’, p = 2
Gaussian NB	Defaults: var smoothing = 1 × 10^−9^

**Table 4:** Classification performance of five machine learning models from the TUG task.

Model	Accuracy	Precision	Recall	F1-Score	ROC-AUC
SVM-RBF	0.813	0.778	0.875	0.824	0.859
LR	0.875	1.000	0.750	0.857	0.922
RF	0.813	0.778	0.875	0.824	0.906
k-NN	0.750	0.833	0.625	0.714	0.781
Gaussian NB	0.625	0.583	0.875	0.700	0.797

**Table 5:** Features are most frequently retained by sequential forward selection across five classifiers.

CoP Features				
No	Name	Selection Models	No. of Models Selected	Category
1	Maximal distance (Radius)—Average	SVM, RF, LR, KNN, GNB	5	Positional
2	Centroidal frequency (Power Spectrum Density) ML—Asymmetry	SVM, RF, LR, GNB	4	Frequency
3	Energy content below 0.5 Hz (Power Spectrum Density) ML—Asymmetry	SVM, RF, KNN, GNB	4	Frequency
4	Mean positive peak velocity AP—Asymmetry	RF, LR, KNN, GNB	4	Dynamic
5	Principal sway direction—Asymmetry	SVM, RF, LR, GNB	4	Positional
6	Frequency Quotient Power Spectrum Density ML—Average	RF, LR, KNN, GNB	4	Frequency
7	Maximal distance AP—Average	SVM, LR, KNN, GNB	4	Positional
8	Mean Velocity AP—Average	SVM, RF, LR, KNN	4	Dynamic
9	Mean Velocity ML-AP—Average	SVM, RF, LR, KNN	4	Dynamic
10	Mean positive peak velocity ML—Average	SVM, LR, KNN, GNB	4	Dynamic
11	95% confidence ellipse area—Asymmetry	RF, LR, KNN	3	Positional
12	Mean Velocity ML-AP—Asymmetry	LR, KNN, GNB	3	Dynamic
13	Mean positive peak velocity ML—Asymmetry	SVM, LR, KNN	3	Dynamic
14	Range ML—Asymmetry	SVM, KNN, GNB	3	Positional
15	Centroidal frequency (Power Spectrum Density) ML—Average	SVM, LR, KNN	3	Frequency
16	Energy content below 0.5 Hz (Power Spectrum Density) AP—Average	SVM, RF, KNN	3	Frequency
17	Mode of Power Spectrum Density ML—Average	SVM, LR, GNB	3	Frequency
18	Frequency Quotient Power Spectrum Density AP—Average	RF, KNN, GNB	3	Frequency
19	Mean frequency ML-AP—Average	SVM, RF, KNN	3	Dynamic
20	Mean Velocity ML—Average	SVM, LR, GNB	3	Dynamic
21	Mean positive peak velocity AP—Average	SVM, LR, GNB	3	Dynamic
22	50% Power Frequency ML—Average	SVM, LR, KNN	3	Frequency
23	Short-term diffusion coefficient AP—Average	SVM, RF, KNN	3	Stochastic
24	Sway area per second ML-AP—Average	LR, KNN, GNB	3	Dynamic

**Table 6:** Classification performance of five machine learning models from the walking-only task.

Model	Accuracy	Precision	Recall	F1-Score	ROC-AUC
SVM-RBF	0.625	0.625	0.625	0.625	0.656
LR	0.625	0.583	0.875	0.700	0.500
RF	0.563	0.545	0.750	0.632	0.602
k-NN	0.688	0.800	0.500	0.615	0.734
Gaussian NB	0.625	0.583	0.875	0.700	0.484

## Data Availability

The datasets used in this study are publicly available via Synapse Storage (https://www.synapse.org/Synapse:syn52540892/wiki/623753, accessed on 13 February 2025).

## Supplementary Materials

The following supporting information can be downloaded at: https://www.mdpi.com/article/10.3390/s25185859/s1, Table S1. Basic features that help to generate Positional features. Table S2. Positional features used for generating new dataset. Table S3. Basic features that help to generate Dynamic features. Table S4. Dynamic features used for generating new dataset. Table S5. Frequency-domain features used for generating new dataset. Table S6. Stochastic features used for generating new dataset. Table S7. Features selected by sequential forward selection for the best-performing model (LR).
