# Supplementary material for: Multi-Domain CoP Feature Analysis of Functional Mobility for Parkinson’s Disease Detection Using Wearable Pressure Insoles
Source: Sensors (Basel). 2025 Sep 19;25(18):5859. doi: 10.3390/s25185859 (PMC12473803; doi:10.3390/s25185859)
Supplement: Supplementary file 1 [file sensors-25-05859-s001.zip › sensors-3833199-supplementary.pdf]

Supplementary Materials

All CoP feature equations were adopted and computed on a per-foot basis over the full duration of each task. The exact mathematical definitions and formulations are described in detail by Quijoux et al. [31], and the open-source implementation provided by the authors was used in this study to ensure methodological consistency.

Table S1: Basic features that help to generate Positional features.

Feature	Equation / Definition
**Centered ML coordinates**	$$X_n = ML_n - \frac{1}{N}\sum_{i=1}^{N} ML_i$$ ▪ ML_n is the original ML CoP value at time index n,<br>▪ N is the total number of samples,<br>▪ X_n is the centered ML coordinate used in most downstream features.
**Centered AP coordinates**	$$Y_n = AP_n - \frac{1}{N}\sum_{i=1}^{N} AP_i$$ ▪ $AP_n$is the original medio-lateral CoP value at time index n,<br>▪ N is the total number of samples,<br>Y_n is the centered ML coordinate used in most downstream features.

Table S2: Positional features used for generating new dataset

No	Feature	Equation / Definition
1	Mean ML	$\frac{1}{N}\sum_{n=1}^{N} ML_n$
2	Mean AP	$\frac{1}{N}\sum_{n=1}^{N} AP_n$
3	Mean distance ML	$\frac{1}{N}\sum_{n=1}^{N} \lvert X_n \rvert$
4	Mean distance AP	$\frac{1}{N}\sum_{n=1}^{N} \lvert Y_n \rvert$
5	Mean distance (Radius)	$\frac{1}{N}\sum_{n=1}^{N} \lvert R_n \rvert$
6	Maximal distance ML	$Max_{1 \le n \le N\ \lvert X_n \rvert}$
7	Maximal distance AP	$Max_{1 \le n \le N\ \lvert Y_n \rvert}$
8	Maximal distance (Radius)	$Max_{1 \le n \le N\ (R_n)}$
9	Root-mean-square ML	$\sqrt{\frac{1}{N}\sum_{n=1}^{N} X_n^2}$
10	Root Mean Square AP	$\sqrt{\frac{1}{N}\sum_{n=1}^{N} Y_n^2}$

11	Root Mean Square (Radius)	$\sqrt{\frac{1}{N}\sum_{n=1}^{N} R_n^2}$
12	Range ML	$Max_{1\le n\le m\le N}\,|X_n - X_m|$<br><br>$X_n$, : CoP position at time step n (centered)<br>X_m: CoP position at a different time step m
13	Range AP	$Max_{1\le n\le m\le N}\,|Y_n - Y_m|$<br><br>$Y_n$, : CoP position at time step n (centered)<br>Y_m: CoP position at a different time step m
14	Range ML-AP	$Max_{1\le n\le m\le N}\sqrt{(X_n - X_m)^2 + (Y_n - Y_m)^2}$
15	Ratio of range ML-AP	$\frac{Range_{ML}}{Range_{AP}}$
16	Planar deviation ML-AP	$\sqrt{{RMS_{ML}}^2 + {RMS_{AP}}^2}$
17	Coefficient of sway direction ML-AP	$\frac{Cov_{(ML,AP)}}{RMS_{ML} \times RMS_{AP}}$
18	95 % confidence ellipse area	$2\pi\frac{N-1}{N-2}.F_{0.95,2,N-2}.\sqrt{RMS_{ML}^2.RMS_{AP}^2 - Cov^2}$<br><br>$F_{0.95,2,N-2}$: 95% quantile of the F-distribution with degrees of freedom
19	Principal sway direction	$arccos\left(\frac{|v_2|}{\sqrt{v_1^2+v_2^2}}\right).\frac{180}{\pi}$<br><br>v = (v_1, v_2): The eigenvector corresponding to the largest eigenvalue from PCA on the CoP data (X_n, Y_n)

Table S3: Basic features that help to generate Dynamic features

Feature	Equation / Definition
Sway length ML	$\sum_{n=1}^{N-1} X_{n+1} - X_n$
Sway length ML	$\sum_{n=1}^{N-1} Y_{n+1} - Y_n$
Total sway length	$\sum_{n=1}^{N-1} \sqrt{(X_{n+1} - X_n)^2 + (Y_{n+1} - Y_n)^2}$

Table S4: Dynamic features used for generating new dataset

No	Feature	Equation / Definition
1	Mean velocity ML	$\frac{\boldsymbol{Sway\ Length_{ML}}}{\boldsymbol{T}}$<br>T: total duration

2	Mean velocity AP	$\frac{Sway\ Length_{AP}}{T}$
3	Mean velocity ML-AP	$\frac{Sway\ Length}{T}$
4	Sway area per second ML-AP	$\frac{1}{2T}\sum_{n=1}^{N-1}\lvert X_{n+1}Y_n - X_nY_{n+1}\rvert$
5	Phase-plane parameter ML	$\sqrt{{RMS_{ML}}^2+STD\ {v_{ML}}^2}$
6	Phase-plane parameter AP	$\sqrt{{RMS_{AP}}^2+STD\ {v_{AP}}^2}$
7	Length over area (LFS)	$\frac{Sway\ Length}{95\%\ Conf\ Area}$
8	Fractal dimension	$\frac{logN}{logN + log\sqrt{\frac{4}{\pi}\times 95\%\ Conf\ Area} - \log(Sway\ Length)}$
9	Zero crossings of velocity ML	$\#Z^{V^X}$
10	Zero crossings of velocity AP	$\#Z^{V^X}$
11	Mean positive peak velocity ML	Average of the positive velocity peaks of the ML velocity signal
12	Mean positive peak velocity AP	Average of the positive velocity peaks of the AP velocity signal
13	Mean negative peak velocity ML	Average of the negative velocity peaks of the ML velocity signal
14	Mean negative peak velocity AP	Average of the negative velocity peaks of the AP velocity signal
15	Mean peak velocity ML	$\frac{1}{K}\sum_{l=1}^{K} p_l^{V^X}$
16	Mean peak velocity AP	$\frac{1}{K}\sum_{l=1}^{K} p_l^{V^Y}$
17	Mean sway density peak	$\frac{1}{K}\sum_{l=1}^{K} p_l^{SD}$<br>$p_l^{SD}$: The distance between two successive peaks
18	Distance between sway-density peaks	Mean inter-peak distance of the sway-density maxima in the CoP plan
19	Mean frequency ML	$\frac{1}{4\sqrt{2}}\times\frac{Mean\ v_{ML}}{Mean\ Dist}$
20	Mean frequency AP	$\frac{1}{4\sqrt{2}}\times\frac{Mean\ v_{AP}}{Mean\ Dist}$
21	Mean frequency ML/AP	$\frac{1}{4\sqrt{2}}\times\frac{Mean\ v}{Mean\ Dist}$

Table S5: Frequency-domain features used for generating new dataset

No.	Feature	Equation / Definition
1	Centroidal frequency (Power Spectrum Density) ML	$\sqrt{\frac{M_2^X}{M_0^X}}$
2	Centroidal frequency (Power Spectrum Density) AP	$\sqrt{\frac{M_2^X}{M_0^X}}$
3	Energy content below 0.5 Hz (Power Spectrum Density) AP	$\sum_{f_{inf}\leq f_k \leq 0.5} \Gamma^X(f_k)$

4	Energy content below 0.5 Hz (Power Spectrum Density) ML	$\sum_{f_{inf} \leq f_k \leq 0.5} \Gamma^X(f_k)$
5	Energy content 0.5–2Hz (Power Spectrum Density) ML	$\sum_{0.5 \leq f_k \leq 2} \Gamma^X(f_k)$
6	Energy content 0.5–2Hz (Power Spectrum Density) ML	$\sum_{0.5 \leq f_k \leq 2} \Gamma^X(f_k)$
7	Energy content above 2 Hz (Power Spectrum Density) AP	$\sum_{2 \leq f_k \leq f_{sup}} (f_k)$
8	Energy content above 2 Hz (Power Spectrum Density) ML	$\sum_{2 \leq f_k \leq f_{sup}} \Gamma^X(f_k)$
9	Frequency dispersion (Power Spectrum Density) ML	$\sqrt{1 - \frac{(M_1^X)^2}{M_2^X M_0^X}}$
10	Frequency dispersion (Power Spectrum Density) AP	$\sqrt{1 - \frac{(M_1^X)^2}{M_2^X M_0^X}}$
11	Mode of Power Spectrum Density ML	$\frac{F_s}{N} \times \arg max_{k_{inf} \leq k \leq k_{sup}} \Gamma_k^X$
12	Mode of Power Spectrum Density AP	$\frac{F_s}{N} \times \arg max_{k_{inf} \leq k \leq k_{sup}} \Gamma_k^X$
13	Frequency quotient Power Spectrum Density ML	$\frac{\sum_{2 \leq f_k \leq 5} \Gamma^X(f_k)}{\sum_{f_{inf} \leq f_k \leq 2} \Gamma^X(f_k)}$
14	Frequency quotient Power Spectrum Density AP	$\frac{\sum_{2 \leq f_k \leq 5} \Gamma^X(f_k)}{\sum_{f_{inf} \leq f_k \leq 2} \Gamma^X(f_k)}$
15	50% Power Frequency ML	Frequency containing 50% of the PSD for ML coordinates
16	50% Power Frequency AP	Frequency containing 50% of the PSD for AP coordinates
17	95% Power Frequency ML	Frequency containing 95% of the PSD for ML coordinates
18	95% Power Frequency AP	Frequency containing 95% of the PSD for AP coordinates
19	Total power ML (Power Spectrum Density)	$\sum_{k=k_{inf}}^{k_{sup}} \Gamma_k^X$
20	Total power AP (Power Spectrum Density)	$\sum_{k=k_{inf}}^{k_{sup}} \Gamma_k^X$

Table S6: Stochastic features used for generating new dataset

No.	Feature	Equation / Definition
1	Long-term diffusion coefficient ML	$exp(\hat{\alpha}_l^X)$
2	Long-term diffusion coefficient AP	$exp(\hat{\alpha}_l^X)$
3	Long-term scaling coefficient ML	$\hat{\beta}_l^X/2$
4	Long-term scaling coefficient AP	$\hat{\beta}_l^X/2$
5	Short-term diffusion coefficient ML	$exp(\hat{\alpha}_s^X)$
6	Short-term diffusion coefficient AP	$exp(\hat{\alpha}_s^X)$
7	Short-term scaling coefficient ML	$\hat{\beta}_s^X/2$
8	Short-term scaling coefficient AP	$\hat{\beta}_s^X/2$
9	Critical time ML	$\exp\left(\frac{\hat{\beta}_s^X - \hat{\beta}_s^X}{\hat{\alpha}_s^X - \hat{\alpha}_l^X}\right)$

10	Critical time AP	$\exp\left(\frac{\hat{\beta}_s^X - \hat{\beta}_s^X}{\hat{\alpha}_s^X - \hat{\alpha}_l^X}\right)$
11	Critical displacement ML	$\hat{\alpha}_s^X \times$ Critical time ML+$\hat{\beta}_s^X$
12	Critical displacement AP	$\hat{\alpha}_s^X \times$ Critical time AP+$\hat{\beta}_s^X$

Table S7: Features selected by sequential forward selection for the best-performing model (LR)

No.	Feature
1	Mode of Power Spectrum Density ML - Average
2	Centroidal frequency (Power Spectrum Density) ML- Asymmetry
3	Maximal distance (Radius) - Average
4	95 % confidence ellipse area - Asymmetry
5	Mean velocity ML - Average
6	Principal sway direction - Asymmetry
7	Mean peak velocity ML - Average
8	Mean velocity ML-AP - Average
9	Sway area per second ML/AP - Average
10	Mean positive peak velocity ML - Average
11	Mean negative peak velocity ML - Average
12	Maximal distance AP - Average
13	Mean positive peak velocity AP - Asymmetry
14	Frequency quotient Power Spectrum Density ML - Average
15	Centroidal frequency (Power Spectrum Density) ML - Average
16	Mean velocity AP - Average
17	50% Power Frequency ML - Average
18	Range AP - Asymmetry
19	Mean positive peak velocity ML - Asymmetry
20	Mean velocity ML-AP - Asymmetry
21	Phase-plane parameter ML - Average
22	Mean positive peak velocity AP - Average
23	95% Power Frequency ML - Average
